# Stereotactic Radiosurgery and Immune Checkpoint Inhibitors in the Management of Brain Metastases

**DOI:** 10.3390/ijms19103054

**Published:** 2018-10-07

**Authors:** Eric J. Lehrer, Heather M. McGee, Jennifer L. Peterson, Laura Vallow, Henry Ruiz-Garcia, Nicholas G. Zaorsky, Sonam Sharma, Daniel M. Trifiletti

**Affiliations:** 1 Department of Radiation Oncology, The Icahn School of Medicine at Mount Sinai, New York, NY 10029, USA; ericjlehrer@gmail.com (E.J.L.); heather.mcgee@mountsinai.org (H.M.M.); sonam.sharma@mountsinai.org (S.S.); 2 Department of Radiation Oncology, Mayo Clinic, Jacksonville, FL 32224, USA; Peterson.Jennifer2@mayo.edu (J.L.P.); Vallow.Laura@mayo.edu (L.V.); RuizGarcia.Henry@mayo.edu (H.R.-G.); 3 Department of Neurosurgery, Mayo Clinic, Jacksonville, FL 32224, USA; 4 Department of Radiation Oncology, Penn State Cancer Institute, Hershey, PA 17033, USA; nicholaszaorsky@gmail.com

**Keywords:** brain metastases, immunotherapy, checkpoint inhibitors, radiation oncology, radiosurgery

## Abstract

Brain metastases traditionally carried a poor prognosis with an overall survival of weeks to months in the absence of treatment. Radiation therapy modalities include whole brain radiation therapy (WBRT) and stereotactic radiosurgery (SRS). WBRT delivers a relatively low dose of radiation, has neurocognitive sequelae, and has not been investigated for its immunostimulatory effects. Furthermore, WBRT exposes the entire intracranial tumor immune microenvironment to radiation. SRS delivers a high dose of conformal radiation with image guidance to minimize dose to surrounding normal brain tissue, and appears to promote anti-tumor immunity. In parallel with many of these discoveries, immune checkpoint inhibitors (ICIs) have demonstrated a survival advantage in multiple malignancies commonly associated with brain metastases (e.g., melanoma). Combination SRS and ICI are theorized to be synergistic in anti-tumor immunity directed to brain metastases. The purpose of this review is to explore the synergy of SRS and ICIs, including pre-clinical data, existing clinical data, and ongoing prospective trials.

## 1. Introduction

It is estimated that 200,000 patients are diagnosed with brain metastases each year in the United States, due to the fact that about 10–30% of cancer patients are diagnosed with brain metastases over the course of their disease [1,2]. However, the true incidence is likely higher, which is due to a combination of factors, such as an increased incidence noted on autopsy and limitations in reporting with national registries (e.g., surveillance, epidemiology, and end results), which tend to focus more on the initial diagnosis and treatment of malignancies rather than events that occur later in the disease course [3,4]. Additionally, ongoing advances in systemic therapies have resulted in improved overall survival with an associated increase in the incidence of brain metastases, largely due to an increase in the number of patients who have systemic disease control with progression only in the central nervous system (CNS).

**Figure 1:**
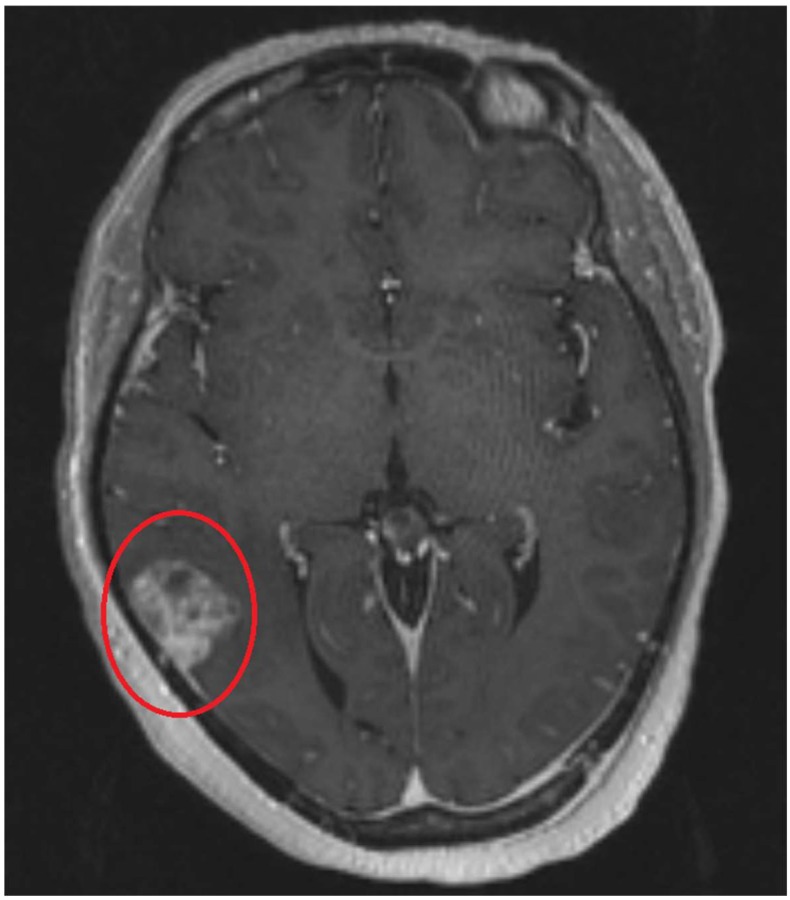
A T1 post-contrast axial magnetic resonance image of a contrast-enhancing tumor (circled in red). This patient is a 57-year-old female with metastatic breast carcinoma. The image and presentation are consistent with a brain metastasis.

Currently, brain metastases (Figure 1) represent the most common intracranial neoplasm and are estimated to occur up to ten times more frequently than primary brain tumors [3]. While the most common primary cancers that metastasize to the brain are non-small cell carcinoma, melanoma, renal cell carcinoma, and breast cancer, there is now an increased number of patients with brain metastases due to less common histologies (e.g., pancreatic cancer), as survival improves for these types of cancers that traditionally had a very poor prognosis [1,5,6]. Historically, the prognosis for brain metastases was poor with a median survival of 3–4 months in patients who were treated non-surgically [7].

For many years, brain metastases were considered a discrete condition with very little attention paid to the underlying biology of each patient’s disease. A paradigm shift occurred in 1997 when a recursive partitioning analysis (RPA) including 1200 patients from three Radiation Therapy Oncology Group studies was published [8]. Median survival time ranged from the best survival of 7.1 months, for patients with a Karnofsky Performance Status (KPS) > 70%, age < 65 years, and a controlled primary tumor with no other site of metastatic disease (RPA class I), to the worst survival of 2.3 months, for patients with a KPS < 70% (RPA class III). All other patients (RPA class II) had a median survival of 4.2 months. From this point forward, individual patient characteristics began to play more of a prominent role in the management of brain metastases. Today, the Diagnosis-Specific Graded Prognostic Assessment (DS-GPA) accounts for the primary tumor type and other unique features in order to take individual patient characteristics into account (e.g., age, KPS, and histology) [9]. However, the DS-GPA is not without limitations, such as it being based on retrospective data involving patients treated between 1985 and 2007. During (and since) that time period, there have been many new advances in treating and staging these patients. Therefore, it is important to use the DS-GPA in the context of individual patient factors.

## 2. Traditional Treatment of Brain Metastases

Traditional treatment strategies for brain metastases involves a complex multimodality regimen, consisting of a combination of surgery, SRS, WBRT, glucocorticoids, or systemic treatment alone. The choice of treatment is often guided by patient specific factors (e.g., DS-GPA and medical comorbidities); however, the optimal way to coordinate these therapies is unknown and prospective data are needed to ascertain the answer to this question. Whole brain radiation therapy had been the mainstay of radiation treatment for decades in these patients. Most commonly, WBRT is delivered over the course of two weeks, to a total dose of 30 Gy in 10 fractions [10,11]. While WBRT has been shown to improve rates of local tumor control (LC), it is not associated with an improvement in overall survival (OS) [12,13,14]. Furthermore, its association with late toxicities and a decrease in quality of life due to neurocognitive decline has led to the development of hippocampal sparing WBRT [15,16,17,18,19,20,21,22]. WBRT remains an excellent treatment option for patients with a large burden of metastatic disease and has the added benefit of targeting microscopic disease that has disseminated to other locations within the brain. 

Patchell et al. authored the original study that demonstrated a benefit with the addition of surgery to WBRT, which included 48 patients with single brain metastases randomized to surgical resection + WBRT versus biopsy only + WBRT [23]. The surgery arm had a decreased rate of local recurrence (20% surgery + WBRT versus 52% WBRT alone); additionally, the surgery arm had improved overall survival (40 weeks versus 15 weeks). Most importantly, patients who received surgery had an improved quality of life with a significantly increased time to remain functionally independent, 38 weeks in the surgery arm versus eight weeks in the WBRT arm [23]. This study showed that surgery combined with WBRT over WBRT alone was associated with a longer time to recurrence, improved overall survival, decreased risk of neurologic death, and increased functional independence. 

Patchell et al. conducted a subsequent trial in 1998, exploring whether surgery alone without WBRT was sufficient [12]. This phase III trial enrolled 95 patients with single brain metastases and MRI-confirmed gross total resection with randomization to surgery +/− WBRT. Local failure at one year was 66% after surgery versus 20% after surgery + WBRT. The distant failure at one year was 50% for the surgery only arm versus 18% for surgery + WBRT. Additionally, there was a decreased risk of neurologic death (44% after surgery alone vs 15% after surgery + WBRT). However, there was no significant difference in median OS with or without the addition of WBRT following surgery [12].

**Figure 2:**
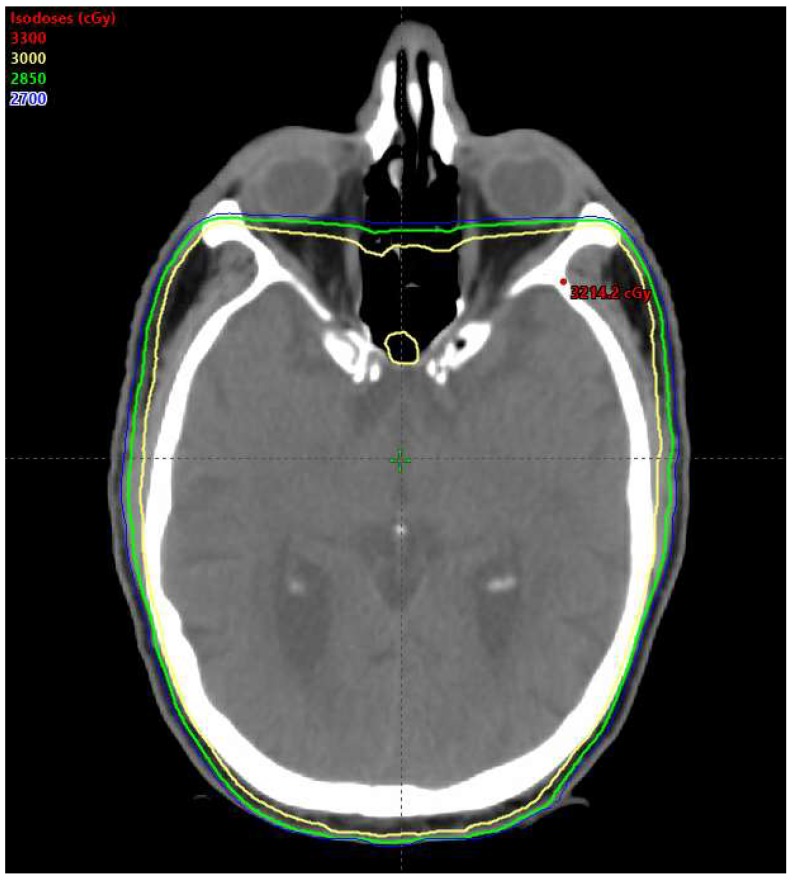
A radiation treatment plan in a patient with multiple brain metastases receiving whole brain radiation therapy to a dose of 30 Gy.

Since the 1990s, there have been multiple prospective randomized trials demonstrating local failure rates of 55–65% with surgical resection alone, and a reduction in both locoregional failure and the incidence of new metastases with WBRT following resection [24,25]. These findings may indicate synergy between surgical resection and WBRT. Figure 2 depicts a patient with brain metastases who was treated with WBRT to a dose of 30 Gy. As depicted, the entire intracranial tumor immune microenvironment is impacted by WBRT.

## 3. Stereotactic Radiosurgery

**Figure 3:**
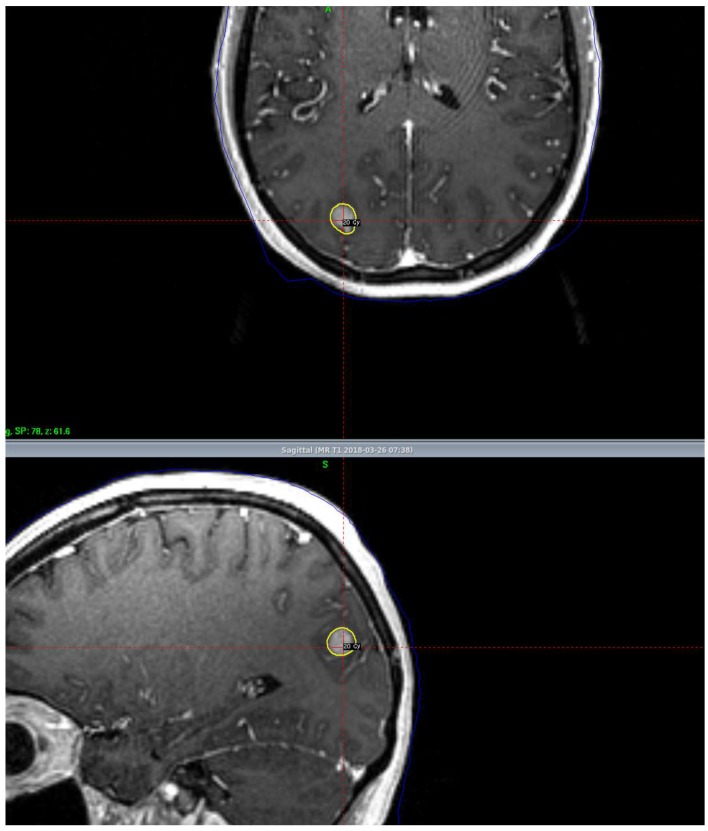
A radiation treatment plan in a patient with a brain metastasis receiving stereotactic radiosurgery to a dose of 20 Gy prescribed to the tumor margin. This patient maintained local control of their disease at one year.

Stereotactic radiosurgery is the delivery of a large dose of highly conformal radiation therapy (RT) in a single session to a specified target while limiting dose to normal tissue, as shown in Figure 3. The original Gamma Knife^®^ was developed in the 1960s by Swedish neurosurgeon Lars Leksell, which uses cobalt sources to deliver RT. Traditionally, delivery of SRS via this platform requires the placement of a stereotactic frame; however, now linear accelerator (LINAC)-based SRS, Cyberknife^®^, and Gamma Knife ICON^®^ technologies are all options to treat patients with “frameless” SRS. SRS offers the option of increased LC without the neurocognitive side effects of WBRT; therefore, SRS has emerged as one of the most effective treatments for brain metastases [16,24,26]. Additionally, it can often be performed in a single session and may not require interruption or delay in initiating systemic therapies, such as immunotherapy. 

## 4. Stereotactic Radiosurgery in the Definitive and Post-Operative Setting

The use of SRS in the definitive setting has been shown to be safe and effective in multiple randomized prospective trials [15,16]. In 2009, Chang et al. published a landmark trial that randomized 58 patients with 1–3 newly-diagnosed brain metastases to receive either SRS or SRS + WBRT [15]. At four months, 64% of patients receiving SRS + WBRT experienced cognitive deterioration, compared to 20% of patients in the SRS arm. As a result of these findings, the trial was stopped early due to the greater cognitive decline in the SRS + WBRT arm [15]. The one-year OS in this study was 63% versus 21%, favoring the SRS arm (*p* = 0.003). However, one-year LC was 67% versus 100%, favoring the SRS + WBRT arm (*p* = 0.012) and one-year distant brain control was 45% versus 73%, favoring the SRS arm (*p* = 0.02) [15].

In 2016, Brown et al. published N0574, which was a multi-institutional phase 3 trial that randomized 213 patients with 1–3 brain metastases across 34 institutions to either SRS alone or SRS + WBRT [16]. Their findings demonstrated less cognitive decline at three months favoring the SRS-only arm (*p* < 0.001) and improved quality of life favoring the SRS arm (*p* = 0.001); however, no survival benefit was observed with a median OS of 10.4 months versus 7.4 months for the SRS and SRS + WBRT arms, respectively. No differences in LC were found at three months; however, the SRS + WBRT arm was eventually shown to have more favorable LC rates at six months (81.6% versus 92.6%) and 12 months (72.8% versus 90.1%) [16]. Since there was no statistically significant difference in overall survival between the groups, this study suggested that SRS may be preferred for patients with one to three brain metastases because it is associated with less cognitive decline.

Two landmark prospective randomized studies were published in 2017, which served to further validate the utility of adding SRS in the post-operative setting [24,25]. The NCCTG (N107C/CEC3) was a multicenter phase 3 trial that randomized patients with resected brain metastases to either SRS alone or WBRT [24]. This study enrolled 194 patients across 48 institutions in the United States and Canada. The cognitive deterioration-free survival time was longer in the SRS only arm than the WBRT arm, 3.7 months versus 3 months (HR: 0.47; *p* < 0.0001); however, a survival benefit was not observed [24]. Additionally, LC and distant brain control favored the WBRT arm (*p* < 0.01) [24]. Mahajan et al. published a single-center phase 3 trial in 2017 that randomized 132 patients who underwent complete surgical resection for 1–3 brain metastases to either observation or post-operative SRS [25]. Local tumor control at one-year was 43% versus 72% in the observation and SRS groups, respectively (*p* = 0.015) [25].

Presently, the National Comprehensive Cancer Network (NCCN) recommends SRS as the preferred treatment option in select groups of patients with brain metastases, particularly ones with a low burden of metastatic disease within the brain [3]. An analysis conducted in 2006 of patients receiving SRS for four or more brain metastases demonstrated that the total tumor volume was the most significant factor associated with survival, rather than the number of metastases. While SRS has been shown to be associated with less cognitive decline in multiple settings, its toxicity profile does include the development of radionecrosis [27]. 

## 5. Impact of Radiation Therapy on the Immune System

Over the past 10–15 years, multiple pre-clinical studies have demonstrated that radiation can be used as an in situ vaccine because it leads to the release of tumor-associated antigens, which activate antigen presenting cells (APC) to migrate to the draining lymph nodes where they prime cytotoxic CD8+ T cells to generate an adaptive immune response [28]. Specifically, ablative doses of RT can increase T cell priming due to cross-presentation of endogenously acquired tumor-derived peptides via the major histocompatibility complex (MHC) class I pathway, which is normally used to present antigens from intracellular pathogens. In a study using subcutaneous murine B16 melanoma and 4T1 mammary carcinoma models, treating tumors with a single fraction of 14–25 Gy led to an increase in T cell priming within the draining lymph nodes and a CD8^+^ T cell mediated decrease in the size of both primary tumors and distant metastatic lesions. [28]. Interestingly, this was not seen after treatment with conventionally fractionated RT [28]. However, other studies have emphasized that hypofractionated radiation regimens (e.g., 8 Gy × 3 fractions) result in a more robust CD8+ T cell-mediated abscopal response and improved survival when compared to high-dose single fraction (e.g., 20 Gy × 1 fraction) in the context of cytotoxic T-lymphocyte-associated protein 4 (CTLA-4) blockade [29]. One explanation for this is that hypofractionated radiation causes double-stranded DNA (dsDNA) to accumulate in the cytoplasm, which stimulates the cGAS/STING (STimulator of INterferon Genes)/IFN-beta pathway to recruit Batf3^+^ dendritic cells (DC) that can activate CD8^+^ T cell-mediated abscopal responses. However, radiation doses > 12 Gy per fraction induce the expression of the DNA exonuclease three prime repair exonuclease 1 (TREX1; 3’ → 5’), which degrades cytosolic dsDNA, abrogating radiation-induced c-GAS/STING induction of Type I interferon (IFN) responses, and leading to a decrease systemic anti-tumor immunity [30]. Immunogenic cell death (ICD), which can occur after radiotherapy, is characterized by the exposure of calreticulin on the surface of dying cells, as well as release of ATP (adenosine triphosphate) and secretion of high-mobility group box 1 protein into the tumor microenvironment [31]. All of these markers of ICD can increase antigen-presentation and subsequent CD8^+^ T cell activation. However, an important caveat is that all of the studies investigating these pathways used subcutaneous murine models. 

Additional evidence suggests that radiation alone also activates many immunosuppressive mechanisms in the tumor microenvironment, such as release of transforming growth factor beta (TGF-β) which causes conversion of CD4^+^ T cells to T regulatory cells (Tregs), and polarization of tumor associated macrophages (TAMs) into an immunosuppressive M2 phenotype. In addition, radiation leads to the release of ATP, which is rapidly catabolized into adenosine in the tumor microenvironment. Local accumulation of extracellular adenosine suppresses DC and effector T cells while promoting proliferation of Tregs [32]. Emerging data suggests that the site which is irradiated may play a role in determining the balance between immune activating and immunosuppressive mechanisms [33].

## 6. Immune Checkpoint Inhibitors

The ability to enhance anti-tumor responses by the immune system offers great promise and has elicited a great deal of enthusiasm, particularly in the development of immune checkpoint inhibitors. In 2011, the U.S. Food and Drug Administration (FDA) approved ipilimumab, a monoclonal antibody against CTLA-4 for use in refractory metastatic melanoma [34,35]. CTLA-4 is a receptor on the surface of T cells that binds to the B7-1 molecule in antigen presenting cells, resulting in the delivery of an inhibitory signal which downregulates T cell activity. In 2014, the FDA approved pembrolizumab and nivolumab, which are monoclonal antibodies against programmed death cell protein 1 (PD-1). PD-1 is a receptor that is present on the surface of T cells that is a member of the immunoglobulin superfamily and binds to programmed death ligand-1 (PD-L1) and PD-L2 to decrease T cell activity. PD-L1 is expressed on a wide repertoire of cells such as tumor cells, macrophages, dendritic cells, and B cells. In 2016, atezolizumab, an inhibitor of PD-L1 became FDA approved for bladder cancer and non-small cell lung cancer (NSCLC). 

Pembrolizumab has been shown to improve OS and progression free survival with fewer side-effects when compared to platinum-based chemotherapy in the treatment of advanced NSCLC with at least 50% PD-L1 expression on tumor cells [36]. Presently, pembrolizumab is FDA approved for melanoma, NSCLC, head and neck cancer, urothelial carcinoma, gastric cancer, cervical cancer, and Hodgkin lymphoma. Nivolumab has also shown promise in the clinical setting; two phase 3 trials published in 2015 demonstrated a survival advantage in patients with advanced NSCLC compared to chemotherapy [37,38]. Nivolumab is presently FDA approved for melanoma, NSCLC, renal cell carcinoma (RCC), Hodgkin lymphoma, head and neck cancer, urothelial carcinoma, colorectal cancer (CRC), hepatocellular carcinoma (HCC), and small cell lung cancer (SCLC) (Table 1).

## 7. Rationale for Combining SRS and Immune Checkpoint Inhibitors in Treating Brain Metastases

The CNS had been viewed as immunologically isolated from the peripheral immune system based on experiments conducted in the early-mid 20th century, where tumors and fetal tissues transplanted into the brain parenchyma escaped rejection, unlike their counterparts transplanted in the periphery [39,40,41]. The prevailing opinion for many years was that the CNS was “immunologically privileged” due to: (1) the presence of the blood-brain barrier (BBB), a highly selective permeability barrier formed by endothelial cells and astroglial cells which regulate the entry of blood-borne metabolites and toxins into the brain [42]; (2) the absence of a readily draining lymphatic system which prevented T cells in the cervical lymph nodes from being exposed to CNS antigens; and (3) the belief that tissue resident macrophages in the CNS (microglia) were ineffective APC due to limited expression of MHC or costimulatory molecules in the CNS parenchyma [43]. Unfortunately, this resulted in decreased efforts within the scientific community to develop systemic therapies targeting metastatic disease in the brain. However, more recent work has suggested that if a robust immune response is generated outside the CNS, the immune system can bypass all three of these barriers [44].

Data from multiple groups has challenged the view of immune privilege by revealing that the CNS is not isolated from the rest of the immune system. Neuroinflammation can alter the vasculature of the BBB in order to allow peripheral immune cells to cross the BBB, microglia can play a key role in orchestrating interactions with other immune cells, and T cells activated in the periphery can detect antigens located in the CNS parenchyma [44]. Activated T cells express Very late antigen-4 (VLA-4) and leukocyte-function-associated antigen-1 (LFA-1), which aid T cell migration across the BBB [45,46]. Additionally, recent evidence suggests there is an alternative route of T cell entry into the CNS that is independent of the BBB, in which T cells can migrate from the blood to the CSF via the choroid plexuses within the ventricles [43]. In addition, the discovery of the lymphatic system draining from the CNS to the cervical lymph nodes has provided further evidence that T cells can be activated by CNS-derived antigens.

The first evidence of antigen-specific T cell responses in the CNS came from studies of the “molecular mimicry hypothesis” of autoimmunity. Immune responses against pathogenic epitopes with similarity to molecules in the CNS can clear the pathogens and lead to T cell mediated responses against similar antigens in the CNS. Original evidence of this hypothesis came from pre-clinical studies in a murine model of multiple sclerosis called experimentally-induced autoimmune encephalitis, where activated T cells specific for components of CNS myelin can be transferred into genetically susceptible mice to trigger CNS inflammation and hindlimb paralysis [47,48].

In addition, activation of the peripheral immune response can lead to systemic production of tumor necrosis factor and nitric oxide, which promote transmigration of macrophages and DC across the BBB in order to serve as APC in the brain [49]. In fact, recent evidence has emerged suggesting that peripherally derived macrophages can engraft the brain and maintain an identity that is distinct from the brain-resident microglia [50]; interestingly, these peripherally-derived APC, but not endogenous APC, appear to play a critical role in the generation of pro-inflammatory T cell responses. Endogenous APC, such as microglia, inhibit proinflammatory T cell responses by expressing PD-L1 and anti-inflammatory molecules, such as indolemine [51,52]. In fact, IFN-γ can induce microglia to express PD-L1, suggesting a mechanism by which anti-PD-L1 antibodies may activate microglia to promote T cell activation [51]. 

Other studies in glioma and glioblastoma have shown that these tumors are highly infiltrated by various cells of the myeloid lineage. Murine models have shown that both microglia and macrophages from the periphery accumulate around synergy, and that these cells have characteristics of both the M1 or M2 macrophages [53,54]. In other models utilizing a GL261 murine glioma, CD206 expression by tumor-infiltrating myeloid-derived suppressor cells are regulated by an autocrine mechanism that involves TGF-β [55]. Other pre-clinical studies using glioblastoma models in mice have shown that activating DCs via the TLR3 agonist (poly I:C) in the tumor-draining lymph node can enhance the anti-tumor immune response to checkpoint blockade and increase survival [56]. Taken together, all of these data suggest that APCs activation is critical for the effectiveness of ICI against tumors in the brain.

Checkpoint inhibitors alone are often ineffective against metastatic disease in the brain. Recently, a pre-clinical study elucidated how immune checkpoint inhibitors work in the intracranial immune microenvironment [57]. In this study, the presence of extracranial tumor was necessary for effective responses by combined anti-CTLA-4 and anti-PD-1 therapy. In the absence of extracranial tumor, melanoma in the brain is able to escape the anti-tumor immune response. Interestingly, synergy between ICI and extracranial tumor enhances CD8^+^ T cell recruitment to the brain by peripheral expansion of effector T cells and upregulation of ICAM-1 and VCAM-1 receptors on blood vessels within the tumor [57]. In essence, this study showed that the concept of molecular mimicry which is popular in the autoimmune field can be extended to explain concepts of anti-tumor immunity in the CNS; anti-PD-1 and anti-CTLA-4 therapies are only effective in eliminating intracranial tumors in the context of extracranial disease which provides a source of tumor antigens in the periphery. Perhaps this explains why systemic immune responses in the peripheral blood are not seen after SRS, as they are after ablative doses of radiation to other sites (e.g., lung and liver) [33]. Radiation alone, delivered to the brain may not be able to generate a systemic immune response in the absence of extracranial disease which provides tumor antigens in the periphery to activate antigen-specific T cells that can cross the BBB and exert their anti-tumor effects in the CNS. 

In addition, to an immunosuppressive microenvironment in the brain, there is evidence that tumors in the CNS induce systemic immune suppression via multiple mechanisms, including secretion of TGF-β and sequestration of lymphocytes in the bone marrow. Using a metastatic model of B16 melanoma in the brain and other peripheral sites, Jackson et al., found that CNS melanomas were found to be more tolerogenic than similarly sized tumors outside the CNS due to dysfunctional tumor-specific T cells, and this occurred secondary to an increase in TGF-β secretion from microglia [58]. In this study, a combination of tumor antigen-specific vaccination and focal radiation therapy reversed T cell tolerance and improved survival of mice with intracranial metastases. Not only did this combination of vaccine and focal radiation improve the ratio of T cell effector cells to T regulatory cells, but it also led to a decrease in TGF-β secretion from microglia. These data suggest that CNS tumors may impair systemic antitumor immunity and consequently accelerate cancer progression both in and outside the CNS, whereas antitumor immunity may be restored by combining vaccination with radiation therapy [58]. 

Another mechanism of systemic immunosuppression by tumors that has been described in pre-clinical models is T-cell lymphopenia which can be due to sequestration of naïve T cells in the bone marrow [59]. When tumors are introduced into the intracranial compartment, T cell sequestration is accompanied by decreased S1P1 expression on T cells. Interestingly, inhibiting S1P1 internalization and reversing the sequestration of T cells in the bone marrow increases the efficacy of immune checkpoint inhibitors [59]. Taken together, these data suggests that there are many levels of cross-talk between the CNS and peripheral immune compartments that can lead to systemic immunosuppression. The clinical implication is that the presence of an intracranial metastasis may actually promote systemic disease progression and reduce the effectiveness of immune checkpoint inhibitors even for peripheral tumors. However, this may explain why combining SRS with ICI holds promise for CNS metastases, as SRS may release CNS antigens for presentation and counteract some of these immunosuppressive processes.

## 8. Existing Clinical Data Supporting Combining SRS with Immune Checkpoint Inhibitors

The general rationale for the efficacy of combined radiation and immune checkpoint strategies is that ICD-inducing agents such as radiation enhance APC activation and T cell priming, and PD-1/PD-L1 inhibitors can then reverse immune exhaustion that occurs after chronic T cell activation. Together, these therapies can work in a synergistic manner to increase anti-tumor immunity, and have been combined in many studies which will be outlined here [60].

Table 2 presents a large number of published studies on combination ICI and SRS in the setting of brain metastases. Of the 17 studies presented, 15 included patients with melanoma [61,62,63,64,65,66,67,68,69,70,71,72,73,74,75], two included patients with NSCLC [76,77], and one study included patients with RCC [77]. The ICI in 8/17 studies was ipilimumab [61,62,64,65,69,71,72,73], while 3/17 studies used anti-PD-1 therapies (such as nivolumab and pembrolizumab), and 6/17 studies used a combination of anti-CTLA-4 and anti-PD-1 agents [66,68,70,74,75,77]. Of these 17 studies, only one was prospective [73], while the remaining 16 were retrospective.

In 2015, Kiess et al. at Memorial Sloan Kettering Cancer Center published a retrospective study evaluating outcomes of patients treated with combined SRS and ipilimumab, where 46 patients with melanoma brain metastases were treated with ipilimumab and SRS to a single fraction of 15–24 Gy [64]. These 46 patients were segmented into three groups: SRS prior to ipilimumab, SRS concurrent with ipilimumab (SRS between consecutive ipilimumab doses or within one month of completing ipilimumab), and SRS after ipilimumab (SRS delivery > 1 month after completing ipilimumab). A survival advantage was observed for patients who received concurrent therapy versus ipilimumab administration > 1 month after SRS delivery, with one-year OS of 65% and 56%, respectively. Interestingly, regional brain control at one-year was 31% in the group that received concurrent therapies, 36 % in the ipilimumab after SRS group, and 8% in the ipilimumab before SRS group. These findings were among the first to indicate that combination SRS and ICI may not only elicit an abscopal-like response within the brain, but that this response may be amplified when SRS is administered prior to or concurrently with ICI. 

A similar retrospective study conducted at the University of Virginia was published by Cohen-Inbar et al. in 2017, and included 46 patients treated with ipilimumab and SRS to a median dose of 20 Gy [69]. Patients were segmented into two groups: (1) SRS concurrent with ipilimumab/ipilimumab following SRS; and (2) SRS administration after completion of ipilimumab. There was a notable difference in one-year OS for the concurrent or ipilimumab after SRS group versus the group that received SRS after ipilimumab, 59% versus 33%, respectively. Additionally, an advantage with local tumor control at one-year was observed for the former compared to the latter, 54.4% versus 16.5%, respectively (*p* = 0.005). However, there was a notable higher incidence of radionecrosis in the first group versus the second group. While these results continue to support an improved response when SRS is administered concurrently with ICI, this sequence of administration may result in an increased risk of experiencing late toxicities, such as radionecrosis. 

The concept of delivering checkpoint inhibitors concurrently with SRS was illustrated in a retrospective study conducted at Johns Hopkins University that compared 70 patients with melanoma, NSCLC, or RCC brain metastases who received ipilimumab and/or pembrolizumab/nivolumab in a concurrent or non-concurrent sequence [77]. The median OS for patients who received SRS after ICI had a median OS of 12 months and those who received SRS prior to ICI had a median OS of 15 months. A survival advantage was observed for the concurrent therapy group with a median OS of 18 months when compared to the SRS after ICI group (*p* = 0.021, HR = 2.64) and SRS prior to ICI group (*p* = 0.002, HR = 3.82). 

The first prospective phase I study combining ipilimumab with RT was conducted at Thomas Jefferson University in 2017 [73]. This study randomized 16 patients to one of two arms: (1) WBRT + ipilimumab; and (2) SRS + ipilimumab. SRS was delivered to a median dose of 24 Gy [73]. Patients were treated with ipilimumab and RT concurrently in both arms. A dose-escalation scheme was utilized where ipilimumab was started at 3 mg/kg and escalated to 10 mg/kg, as tolerated. The primary endpoint was to determine the maximum tolerated dose of ipilimumab in combination with both forms of radiation. The majority of toxicities reported were self-limited (grade 1–2) with minimal grade 3 toxicities, no grade 4 toxicities, and no reported development of radionecrosis. However, of the 16 patients enrolled, 14/16 developed disease progression and/or died during the follow-up period, and 9/11 of patients in the SRS arm developed progression or died. Ultimately, the WBRT arm was closed early due to slow accrual. While this study did demonstrate that combination ipilimumab and SRS may be well-tolerated, further prospective data are needed to validate the efficacy and safety of this treatment combination. This also raises the question of why the retrospective data have shown significantly better outcomes, and if there has been significant selection bias in the retrospective studies that may have influenced the outcomes? 

A recently published meta-analysis by Lehrer et al. demonstrated a 5.3% radionecrosis incidence rate for the studies reporting, which was more pronounced in patients receiving ipilimumab over pembrolizumab or nivolumab [78]. Additionally, the same analysis demonstrated a one-year OS of 64.6% versus 51.6% for concurrent and non-concurrent therapy, respectively (*p* < 0.001), which was most commonly defined as SRS and ICI administration within four weeks of one another [78].

## 9. Planned and Ongoing Prospective Randomized Control Trials Assessing the Safety and Efficacy of Combination Therapy with SRS and Immune Checkpoint Inhibitors

While there is a great paucity of prospective data validating the use of SRS combined with ICI in the treatment of brain metastases, there are a several phase 1 and 2 prospective trials planned and underway (Table 3). NCT02696993 is a two-phase study recruiting patients with NSCLC brain metastases. The first phase will assess dose escalation and determine the maximum tolerated dose by comparing nivolumab + SRS and nivolumab + WBRT. The second phase will then assess dose expansion by adding ipilimumab to both arms. The Australian ABC-X Study (NCT03340129) will compare combination nivolumab and ipilimumab; however, this will be in the setting of metastatic melanoma and SRS will be added if progression occurs with dual ICI therapy.

## 10. Conclusions

Patients with brain metastases have greater outcomes than ever before due to the evolution of surgical and SRS techniques alongside improved systemic treatment. Just as the use of SRS involves careful consideration of size, location, mass effect, edema, symptomatic burden, and the extent of visceral disease, there is a need to investigate how all of these factors also influence the clinical response to ICI. While ablative doses of RT, such as those administered with SRS appear to enhance the anti-tumor activity of the immune system, there is less evidence for this in the brain as compared to other organs. Recent discoveries challenging the belief that the brain is “immunologically privileged” have led to increased enthusiasm in combining SRS with immune checkpoint inhibitors. Combination therapy appears to enhance survival and abscopal-like responses within the brain, which may be amplified when administered concurrently rather than sequentially. Ongoing and planned prospective trials are needed to further explore and validate these findings. Future studies will explore elucidating predictive biomarkers that can be utilized to stratify patients based on the likelihood of the response to such combined treatment approaches, but this will require collaboration between immunologists, oncologists, neurosurgeons, and radiation oncologists. 

## Figures and Tables

**Table 1:** Immune checkpoint inhibitors and indications.

Drug	Target	FDA Approved Indications
Ipilimumab	CTLA-4	Metastatic Melanoma
Pembrolizumab	PD-1	Metastatic Melanoma, NSCLC, Head and Neck Cancer, Hodgkin Lymphoma, Urothelial Carcinoma, Gastric Cancer, Cervical Cancer
Nivolumab	PD-1	Metastatic Melanoma, NSCLC, RCC, Hodgkin Lymphoma, Head and Neck Cancer, Urothelial Carcinoma, CRC, HCC, SCLC
Atezolizumab	PD-L1	Bladder Cancer, NSCLC
Ipilimumab + Nivolumab	CTLA-4 + PD-1	RCC, CRC

**Table 2:** Selected studies combining SRS with immune checkpoint inhibitors in brain metastases.

Study	H	Arm	N	ICI Target	DS-GPA	1-Year OS (%)	1-Year LC (%)	1-Year RBC (%)	RN
Mathew et al., 2013 [61].	M	NR	25	CTLA-4	NR	32.6	40	16	NR
Silk et al., 2013 [62].	M	NR	17	CTLA-4	25% (0-1); 39.3% (2); 25% (3); 10.7% (4)	82.3	NR	NR	NR
Ahmed et al., 2016 [63].	M	NR	26	PD-1	27% (1-2); 19% (3-4)	74.7	82	45.9	NR
Kiess et al., 2015 [64].	M	SRS → ICI	19	CTLA-4	3 (median)	56	87	36	NR
		SRS = ICI	15			65	100	31	NR
		ICI → SRS	12			50	89	8	NR
Schoenfeld et al., 2015 [65].	M	SRS → ICI	5	CTLA-4	NR	NR	NR	NR	NR
		SRS = ICI	4			NR	NR	NR	NR
		ICI → SRS	7			NR	NR	NR	NR
Qian et al., 2015 [66].	M	SRS ≠ ICI	22	CTLA-4 or PD-1	3 (median)	44.4	NR	NR	NR
		SRS = ICI	33		2 (median)	62.5	NR	NR	NR
Ahmed et al., 2017 [76].	NSCLC	NR	17	PD-1	59% (0-1.5); 41% (2-3)	40.0	96.0	0.0	NR
Anderson et al., 2017 [67].	M	NR	11	PD-1	3 (median)	NR	NR	NR	0
Choong et al., 2017 [68].	M	NR	39	CTLA-4 or PD-1	NR	54.9	NR	NR	5
Cohen-Inbar et al., 2017 [69].	M	SRS = ICI; SRS → ICI	32	CTLA-4	2.5% (0-1); 53% (2); 18.8% (3); 15.6% (4)	59.0	54.4	25.8	31
		ICI → SRS	14		14.3% (0-1); 64.3% (2); 0% (3); 21.4% (4)	33.0	16.5	26.8	7
Gaudy-Marqueste et al., 2017 [70].	M	SRS → ICI	43	CTLA-4 or PD-1	NR	52.4	NR	NR	NR
Patel et al., 2017 [71].	M	NR	20	CTLA-4	10% (1); 35% (2); 30% (3); 25% (4)	37.1	71.4	12.7	NR
Skrepnik et al., 2017 [72].	M	NR	25	CTLA-4	NR	83.0	94.8	72.0	12
Williams et al., 2017 [73].	M	NR	11	CTLA-4	NR	60.0	NR	NR	0
Yusuf et al., 2017 [75].	M	SRS = ICI	12	CTLA-4 or PD-1	NR	45.0	87.6	46.4	2
		SRS ≠ ICI	6			21.5	NR	0.0	0
Acharya et al., 2017 [74].	M	NR	18	CTLA-4 and/or PD-1	6% (1); 28% (2); 39% (3); 0% (4)	58.5	85.0	60.0	NR
Chen et al., 2018 [77].	M, NSCLC, RCC	SRS → ICI	30	CTLA-4 and/or PD-1	NR	63.6	NR	NR	NR
		SRS = ICI	28			77.9	88.0	NR	NR
		ICI → SRS	23			50.7	NR	NR	NR

Note: ICI → SRS indicates ICI was administered prior to SRS; SRS = ICI indicates that ICI was administered concurrently with SRS; SRS → ICI indicates that ICI was administered after SRS; SRS ≠ ICI indicates that SRS was not administered concurrently with SRS but the relative timing of each treatment was not provided; Radionecrosis (RN) expressed as number of lesions affected

**Table 3:** Active and planned randomized control trials assessing the safety and efficacy of SRS and immune checkpoint inhibitors in the treatment of brain metastases.

Study	Phase	Country	Histology	SRS Dose	ICI Target	Primary Outcome
NCT02886585	2	USA	Melanoma	NR	PD-1	Overall Response Rate; Overall Survival; Extracranial Overall Response Rate
NCT02858869	1	USA	Melanoma and NSCLC	30 Gy/5 fractions 27 Gy/3 fractions 18-21 Gy/1 fraction	PD-1	Dose-Limiting Toxicities
NCT02978404	2	Canada	NSCLC and RCC	15-20 Gy/1 fraction	PD-1	Progression-Free Survival
NCT02696993	1 & 2	USA	NSCLC	NR	CTLA-4 and PD-1	Maximum Tolerated Dose; Dose-Limiting Toxicities
NCT03340129	2	Australia	Melanoma	16-22 Gy/1 fraction	CTLA-4 and PD-1	Intracranial Response to Immune Checkpoint Inhibitor
NCT02716948	1	USA	Melanoma	NR	PD-1	Incidence of Severe Adverse Effects
NCT02097732	2	USA	Melanoma	NR	CTLA-4	Local Control at 6 months
NCT01703507	1	USA	Melanoma	NR	CTLA-4	Maximum Tolerated Dose

## Abbreviations

MDPI	Multidisciplinary Digital Publishing Institute
DOAJ	Directory of open access journals
WBRT	Whole brain radiation therapy
SRS	Stereotactic radiosurgery
ICI	Immune checkpoint inhibitors
CNS	Central nervous system
RPA	Recursive partitioning analysis
KPS	Karnofsky Performance Status
DS-GPA	Diagnosis Specific–Graded Prognostic Assessment
Gy	Gray
LC	Local control
OS	Overall survival
MRI	Magnetic resonance imaging
RT	Radiation therapy
LINAC	Linear accelerator
NCCN	National Comprehensive Cancer Network
APC	Antigen presenting cells
MHC	Major histocompatibility complex
CTLA-4	Cytotoxic T-lymphocyte-associated protein 4
dsDNA	Double-stranded deoxyribonucleic acid
STING	STimulator of INterferon Genes
DC	Dendritic cells
IFN	Interferon
ICD	Immunogenic cell death
ATP	Adenosine triphosphate
TGF-β	Transforming growth factor β
Tregs	T regulatory cells
TAMs	Tumor associated macrophages
DNA	Deoxyribonucleic acid
FDA	Food and Drug Administration
PD-1	Programmed death cell protein 1
PD-L1	Programmed death ligand-1
NSCLC	Non-small cell lung cancer
RCC	Renal cell carcinoma
CRC	Colorectal cancer
HCC	Hepatocellular carcinoma
SCLC	Small cell lung cancer
BBB	Blood brain barrier
VLA-4	Very late antigen-4
LFA-1	Leukocyte-function-associated antigen-1
H	Histology
M	Melanoma
N	Total number of patients
NR	Not reported
USA	United States of America

## References

[1] Barnholtz-Sloan J.S., Sloan A.E., Davis F.G., Vigneau F.D., Lai P., Sawaya R.E. (2004). Incidence proportions of brain metastases in patients diagnosed (1973 to 2001) in the metropolitan detroit cancer surveillance system. J. Clin. Oncol..

[2] Kohler B.A., Ward E., McCarthy B.J., Schymura M.J., Ries L.A., Eheman C., Jemal A., Anderson R.N., Ajani U.A., Edwards B.K. (2011). Annual report to the nation on the status of cancer, 1975–2007, featuring tumors of the brain and other nervous system. J. Natl. Cancer Inst..

[3] Nabors L.B., Portnow J., Ammirati M., Baehring J., Brem H., Butowski N., Fenstermaker R.A., Forsyth P., Hattangadi-Gluth J., Holdhoff M. (2017). Nccn practice guidelines in oncology—Central nervous system cancers, version 1.2017. J. Natl. Compr. Canc. Netw..

[4] Arvold N.D., Lee E.Q., Mehta M.P., Margolin K., Alexander B.M., Lin N.U., Anders C.K., Soffietti R., Camidge D.R., Vogelbaum M.A. (2016). Updates in the management of brain metastases. Neuro. Oncol..

[5] Sanghvi S.M., Lischalk J.W., Cai L., Collins S., Nair M., Collins B., Unger K. (2017). Clinical outcomes of gastrointestinal brain metastases treated with radiotherapy. Radiat. Oncol..

[6] Trifiletti D.M., Patel N., Lee C.C., Romano A.M., Sheehan J.P. (2015). Stereotactic radiosurgery in the treatment of brain metastases from gastrointestinal primaries. J. Neurooncol..

[7] Nieder C., Spanne O., Mehta M.P., Grosu A.L., Geinitz H. (2011). Presentation, patterns of care, and survival in patients with brain metastases: What has changed in the last 20 years?. Cancer.

[8] Gaspar L., Scott C., Rotman M., Asbell S., Phillips T., Wasserman T., McKenna W.G., Byhardt R. (1997). Recursive partitioning analysis (rpa) of prognostic factors in three radiation therapy oncology group (rtog) brain metastases trials. Int. J. Radiat. Oncol. Biol. Phys..

[9] Sperduto P.W., Kased N., Roberge D., Xu Z., Shanley R., Luo X., Sneed P.K., Chao S.T., Weil R.J., Suh J. (2012). Summary report on the graded prognostic assessment: An accurate and facile diagnosis-specific tool to estimate survival for patients with brain metastases. J. Clin. Oncol..

[10] Borgelt B., Gelber R., Kramer S., Brady L.W., Chang C.H., Davis L.W., Perez C.A., Hendrickson F.R. (1980). The palliation of brain metastases: Final results of the first two studies by the radiation therapy oncology group. Int. J. Radiat. Oncol. Biol. Phys..

[11] Sneed P.K., Larson D.A., Wara W.M. (1996). Radiotherapy for cerebral metastases. Neurosurg. Clin. N. Am..

[12] Patchell R.A., Tibbs P.A., Regine W.F., Dempsey R.J., Mohiuddin M., Kryscio R.J., Markesbery W.R., Foon K.A., Young B. (1998). Postoperative radiotherapy in the treatment of single metastases to the brain: A randomized trial. JAMA.

[13] McPherson C.M., Suki D., Feiz-Erfan I., Mahajan A., Chang E., Sawaya R., Lang F.F. (2010). Adjuvant whole-brain radiation therapy after surgical resection of single brain metastases. Neuro. Oncol..

[14] Kocher M., Soffietti R., Abacioglu U., Villa S., Fauchon F., Baumert B.G., Fariselli L., Tzuk-Shina T., Kortmann R.D., Carrie C. (2011). Adjuvant whole-brain radiotherapy versus observation after radiosurgery or surgical resection of one to three cerebral metastases: Results of the eortc 22952–26001 study. J. Clin. Oncol..

[15] Chang E.L., Wefel J.S., Hess K.R., Allen P.K., Lang F.F., Kornguth D.G., Arbuckle R.B., Swint J.M., Shiu A.S., Maor M.H. (2009). Neurocognition in patients with brain metastases treated with radiosurgery or radiosurgery plus whole-brain irradiation: A randomised controlled trial. Lancet Oncol..

[16] Brown P.D., Jaeckle K., Ballman K.V., Farace E., Cerhan J.H., Anderson S.K., Carrero X.W., Barker F.G., Deming R., Burri S.H. (2016). Effect of radiosurgery alone vs radiosurgery with whole brain radiation therapy on cognitive function in patients with 1 to 3 brain metastases: A randomized clinical trial. JAMA.

[17] Brown P.D., Pugh S., Laack N.N., Wefel J.S., Khuntia D., Meyers C., Choucair A., Fox S., Suh J.H., Roberge D. (2013). Memantine for the prevention of cognitive dysfunction in patients receiving whole-brain radiotherapy: A randomized, double-blind, placebo-controlled trial. Neuro. Oncol..

[18] Cohen-Inbar O., Melmer P., Lee C.C., Xu Z., Schlesinger D., Sheehan J.P. (2016). Leukoencephalopathy in long term brain metastases survivors treated with radiosurgery. J. Neurooncol..

[19] McTyre E., Scott J., Chinnaiyan P. (2013). Whole brain radiotherapy for brain metastasis. Surg. Neurol. Int..

[20] Abe E., Aoyama H. (2012). The role of whole brain radiation therapy for the management of brain metastases in the era of stereotactic radiosurgery. Curr. Oncol. Rep..

[21] Duan L., Zeng R., Yang K.H., Tian J.H., Wu X.L., Dai Q., Niu X.D., Ma D.W. (2014). Whole brain radiotherapy combined with stereotactic radiotherapy versus stereotactic radiotherapy alone for brain metastases: A meta-analysis. Asian Pac. J. Cancer Prev..

[22] Gondi V., Pugh S.L., Tome W.A., Caine C., Corn B., Kanner A., Rowley H., Kundapur V., DeNittis A., Greenspoon J.N. (2014). Preservation of memory with conformal avoidance of the hippocampal neural stem-cell compartment during whole-brain radiotherapy for brain metastases (rtog 0933): A phase ii multi-institutional trial. J. Clin. Oncol..

[23] Patchell R.A., Tibbs P.A., Walsh J.W., Dempsey R.J., Maruyama Y., Kryscio R.J., Markesbery W.R., Macdonald J.S., Young B. (1990). A randomized trial of surgery in the treatment of single metastases to the brain. N. Engl. J. Med..

[24] Brown P.D., Ballman K.V., Cerhan J.H., Anderson S.K., Carrero X.W., Whitton A.C., Greenspoon J., Parney I.F., Laack N.N.I., Ashman J.B. (2017). Postoperative stereotactic radiosurgery compared with whole brain radiotherapy for resected metastatic brain disease (ncctg n107c/cec.3): A multicentre, randomised, controlled, phase 3 trial. Lancet Oncol..

[25] Mahajan A., Ahmed S., McAleer M.F., Weinberg J.S., Li J., Brown P., Settle S., Prabhu S.S., Lang F.F., Levine N. (2017). Post-operative stereotactic radiosurgery versus observation for completely resected brain metastases: A single-centre, randomised, controlled, phase 3 trial. Lancet Oncol..

[26] Andrews D.W., Scott C.B., Sperduto P.W., Flanders A.E., Gaspar L.E., Schell M.C., Werner-Wasik M., Demas W., Ryu J., Bahary J.P. (2004). Whole brain radiation therapy with or without stereotactic radiosurgery boost for patients with one to three brain metastases: Phase iii results of the rtog 9508 randomised trial. Lancet.

[27] Chao S.T., Ahluwalia M.S., Barnett G.H., Stevens G.H., Murphy E.S., Stockham A.L., Shiue K., Suh J.H. (2013). Challenges with the diagnosis and treatment of cerebral radiation necrosis. Int. J. Radiat. Oncol. Biol. Phys..

[28] Lee Y., Auh S.L., Wang Y., Burnette B., Wang Y., Meng Y., Beckett M., Sharma R., Chin R., Tu T. (2009). Therapeutic effects of ablative radiation on local tumor require cd8+ T cells: Changing strategies for cancer treatment. Blood.

[29] Dewan M.Z., Galloway A.E., Kawashima N., Dewyngaert J.K., Babb J.S., Formenti S.C., Demaria S. (2009). Fractionated but not single-dose radiotherapy induces an immune-mediated abscopal effect when combined with anti-ctla-4 antibody. Clin. Cancer Res..

[30] Vanpouille-Box C., Alard A., Aryankalayil M.J., Sarfraz Y., Diamond J.M., Schneider R.J., Inghirami G., Coleman C.N., Formenti S.C., Demaria S. (2017). DNA exonuclease trex1 regulates radiotherapy-induced tumour immunogenicity. Nat. Commun..

[31] Golden E.B., Pellicciotta I., Demaria S., Barcellos-Hoff M.H., Formenti S.C. (2012). The convergence of radiation and immunogenic cell death signaling pathways. Front Oncol..

[32] Wennerberg E., Lhuillier C., Vanpouille-Box C., Pilones K.A., Garcia-Martinez E., Rudqvist N.P., Formenti S.C., Demaria S. (2017). Barriers to radiation-induced in situ tumor vaccination. Front Immunol..

[33] McGee H.M., Daly M.E., Azghadi S., Stewart S.L., Oesterich L., Schlom J., Donahue R., Schoenfeld J.D., Chen Q., Rao S. (2018). Stereotactic ablative radiation therapy induces systemic differences in peripheral blood immunophenotype dependent on irradiated site. Int. J. Radiat. Oncol. Biol. Phys..

[34] Hodi F.S., O’Day S.J., McDermott D.F., Weber R.W., Sosman J.A., Haanen J.B., Gonzalez R., Robert C., Schadendorf D., Hassel J.C. (2010). Improved survival with ipilimumab in patients with metastatic melanoma. N. Engl. J. Med..

[35] Robert C., Thomas L., Bondarenko I., O’Day S., Weber J., Garbe C., Lebbe C., Baurain J.F., Testori A., Grob J.J. (2011). Ipilimumab plus dacarbazine for previously untreated metastatic melanoma. N. Engl. J. Med..

[36] Reck M., Rodriguez-Abreu D., Robinson A.G., Hui R., Csoszi T., Fulop A., Gottfried M., Peled N., Tafreshi A., Cuffe S. (2016). Pembrolizumab versus chemotherapy for pd-l1-positive non-small-cell lung cancer. N. Engl. J. Med..

[37] Brahmer J., Reckamp K.L., Baas P., Crino L., Eberhardt W.E., Poddubskaya E., Antonia S., Pluzanski A., Vokes E.E., Holgado E. (2015). Nivolumab versus docetaxel in advanced squamous-cell non-small-cell lung cancer. N. Engl. J. Med..

[38] Borghaei H., Paz-Ares L., Horn L., Spigel D.R., Steins M., Ready N.E., Chow L.Q., Vokes E.E., Felip E., Holgado E. (2015). Nivolumab versus docetaxel in advanced nonsquamous non-small-cell lung cancer. N. Engl. J. Med..

[39] Murphy J.B., Sturm E. (1923). Conditions determining the transplantability of tissues in the brain. J. Exp. Med..

[40] Widner H., Brundin P. (1988). Immunological aspects of grafting in the mammalian central nervous system. A review and speculative synthesis. Brain Res..

[41] Medawar P.B. (1948). Immunity to homologous grafted skin; the fate of skin homografts transplanted to the brain, to subcutaneous tissue, and to the anterior chamber of the eye. Br. J. Exp. Pathol..

[42] Han H.S., Suk K. (2005). The function and integrity of the neurovascular unit rests upon the integration of the vascular and inflammatory cell systems. Curr. Neurovasc. Res..

[43] Ransohoff R.M., Kivisakk P., Kidd G. (2003). Three or more routes for leukocyte migration into the central nervous system. Nat. Rev. Immunol..

[44] Carson M.J., Doose J.M., Melchior B., Schmid C.D., Ploix C.C. (2006). Cns immune privilege: Hiding in plain sight. Immunol. Rev..

[45] Stoll G., Jander S., Schroeter M. (2002). Detrimental and beneficial effects of injury-induced inflammation and cytokine expression in the nervous system. Adv. Exp. Med. Biol..

[46] Carman C.V., Springer T.A. (2004). A transmigratory cup in leukocyte diapedesis both through individual vascular endothelial cells and between them. J. Cell. Biol..

[47] Fujinami R.S., Oldstone M.B. (1985). Amino acid homology between the encephalitogenic site of myelin basic protein and virus: Mechanism for autoimmunity. Science.

[48] Levin M.C., Lee S.M., Kalume F., Morcos Y., Dohan F.C., Hasty K.A., Callaway J.C., Zunt J., Desiderio D., Stuart J.M. (2002). Autoimmunity due to molecular mimicry as a cause of neurological disease. Nat. Med..

[49] Deli M.A., Abraham C.S., Kataoka Y., Niwa M. (2005). Permeability studies on in vitro blood-brain barrier models: Physiology, pathology, and pharmacology. Cell. Mol. Neurobiol..

[50] Cronk J.C., Filiano A.J., Louveau A., Marin I., Marsh R., Ji E., Goldman D.H., Smirnov I., Geraci N., Acton S. (2018). Peripherally derived macrophages can engraft the brain independent of irradiation and maintain an identity distinct from microglia. J. Exp. Med..

[51] Magnus T., Schreiner B., Korn T., Jack C., Guo H., Antel J., Ifergan I., Chen L., Bischof F., Bar-Or A. (2005). Microglial expression of the b7 family member b7 homolog 1 confers strong immune inhibition: Implications for immune responses and autoimmunity in the cns. J. Neurosci..

[52] Kwidzinski E., Bunse J., Aktas O., Richter D., Mutlu L., Zipp F., Nitsch R., Bechmann I. (2005). Indolamine 2,3-dioxygenase is expressed in the cns and down-regulates autoimmune inflammation. FASEB J..

[53] Szulzewsky F., Pelz A., Feng X., Synowitz M., Markovic D., Langmann T., Holtman I.R., Wang X., Eggen B.J., Boddeke H.W. (2015). Glioma-associated microglia/macrophages display an expression profile different from m1 and m2 polarization and highly express gpnmb and spp1. PLoS ONE.

[54] Gabrusiewicz K., Rodriguez B., Wei J., Hashimoto Y., Healy L.M., Maiti S.N., Thomas G., Zhou S., Wang Q., Elakkad A. (2016). Glioblastoma-infiltrated innate immune cells resemble m0 macrophage phenotype. JCI Insight.

[55] Umemura N., Saio M., Suwa T., Kitoh Y., Bai J., Nonaka K., Ouyang G.F., Okada M., Balazs M., Adany R. (2008). Tumor-infiltrating myeloid-derived suppressor cells are pleiotropic-inflamed monocytes/macrophages that bear m1- and m2-type characteristics. J. Leukoc. Biol..

[56] Garzon-Muvdi T., Theodros D., Luksik A.S., Maxwell R., Kim E., Jackson C.M., Belcaid Z., Ganguly S., Tyler B., Brem H. (2018). Dendritic cell activation enhances anti-pd-1 mediated immunotherapy against glioblastoma. Oncotarget.

[57] Taggart D., Andreou T., Scott K.J., Williams J., Rippaus N., Brownlie R.J., Ilett E.J., Salmond R.J., Melcher A., Lorger M. (2018). Anti-pd-1/anti-ctla-4 efficacy in melanoma brain metastases depends on extracranial disease and augmentation of cd8(+) t cell trafficking. Proc. Natl. Acad. Sci. USA.

[58] Jackson C.M., Kochel C.M., Nirschl C.J., Durham N.M., Ruzevick J., Alme A., Francica B.J., Elias J., Daniels A., Dubensky T.W. (2016). Systemic tolerance mediated by melanoma brain tumors is reversible by radiotherapy and vaccination. Clin. Cancer Res..

[59] Chongsathidkiet P., Jackson C., Koyama S., Loebel F., Cui X., Farber S.H., Woroniecka K., Elsamadicy A.A., Dechant C.A., Kemeny H.R. (2018). Sequestration of t cells in bone marrow in the setting of glioblastoma and other intracranial tumors. Nat. Med..

[60] Formenti S.C., Demaria S. (2018). Understanding responses to stereotactic body radiotherapy and pembrolizumab. J. Clin. Oncol..

[61] Mathew M., Tam M., Ott P.A., Pavlick A.C., Rush S.C., Donahue B.R., Golfinos J.G., Parker E.C., Huang P.P., Narayana A. (2013). Ipilimumab in melanoma with limited brain metastases treated with stereotactic radiosurgery. Melanoma Res..

[62] Silk A.W., Bassetti M.F., West B.T., Tsien C.I., Lao C.D. (2013). Ipilimumab and radiation therapy for melanoma brain metastases. Cancer Med..

[63] Ahmed K.A., Stallworth D.G., Kim Y., Johnstone P.A., Harrison L.B., Caudell J.J., Yu H.H., Etame A.B., Weber J.S., Gibney G.T. (2016). Clinical outcomes of melanoma brain metastases treated with stereotactic radiation and anti-pd-1 therapy. Ann. Oncol..

[64] Kiess A.P., Wolchok J.D., Barker C.A., Postow M.A., Tabar V., Huse J.T., Chan T.A., Yamada Y., Beal K. (2015). Stereotactic radiosurgery for melanoma brain metastases in patients receiving ipilimumab: Safety profile and efficacy of combined treatment. Int. J. Radiat. Oncol. Biol. Phys..

[65] Schoenfeld J.D., Mahadevan A., Floyd S.R., Dyer M.A., Catalano P.J., Alexander B.M., McDermott D.F., Kaplan I.D. (2015). Ipilmumab and cranial radiation in metastatic melanoma patients: A case series and review. J. Immunother. Cancer.

[66] Qian J.M., Yu J.B., Kluger H.M., Chiang V.L. (2016). Timing and type of immune checkpoint therapy affect the early radiographic response of melanoma brain metastases to stereotactic radiosurgery. Cancer.

[67] Anderson E.S., Postow M.A., Wolchok J.D., Young R.J., Ballangrud A., Chan T.A., Yamada Y., Beal K. (2017). Melanoma brain metastases treated with stereotactic radiosurgery and concurrent pembrolizumab display marked regression; efficacy and safety of combined treatment. J. Immunother. Cancer.

[68] Choong E.S., Lo S., Drummond M., Fogarty G.B., Menzies A.M., Guminski A., Shivalingam B., Clarke K., Long G.V., Hong A.M. (2017). Survival of patients with melanoma brain metastasis treated with stereotactic radiosurgery and active systemic drug therapies. Eur. J. Cancer.

[69] Cohen-Inbar O., Shih H.H., Xu Z., Schlesinger D., Sheehan J.P. (2017). The effect of timing of stereotactic radiosurgery treatment of melanoma brain metastases treated with ipilimumab. J. Neurosurg..

[70] Gaudy-Marqueste C., Dussouil A.S., Carron R., Troin L., Malissen N., Loundou A., Monestier S., Mallet S., Richard M.A., Regis J.M. (2017). Survival of melanoma patients treated with targeted therapy and immunotherapy after systematic upfront control of brain metastases by radiosurgery. Eur. J. Cancer.

[71] Patel K.R., Shoukat S., Oliver D.E., Chowdhary M., Rizzo M., Lawson D.H., Khosa F., Liu Y., Khan M.K. (2017). Ipilimumab and stereotactic radiosurgery versus stereotactic radiosurgery alone for newly diagnosed melanoma brain metastases. Am. J. Clin. Oncol..

[72] Skrepnik T., Sundararajan S., Cui H., Stea B. (2017). Improved time to disease progression in the brain in patients with melanoma brain metastases treated with concurrent delivery of radiosurgery and ipilimumab. Oncoimmunology.

[73] Williams N.L., Wuthrick E.J., Kim H., Palmer J.D., Garg S., Eldredge-Hindy H., Daskalakis C., Feeney K.J., Mastrangelo M.J., Kim L.J. (2017). Phase 1 study of ipilimumab combined with whole brain radiation therapy or radiosurgery for melanoma patients with brain metastases. Int. J. Radiat. Oncol. Biol. Phys..

[74] Acharya S., Mahmood M., Mullen D., Yang D., Tsien C.I., Huang J., Perkins S.M., Rich K., Chicoine M., Leuthardt E. (2017). Distant intracranial failure in melanoma brain metastases treated with stereotactic radiosurgery in the era of immunotherapy and targeted agents. Adv. Radiat. Oncol..

[75] Yusuf M.B., Amsbaugh M.J., Burton E., Chesney J., Woo S. (2017). Peri-srs administration of immune checkpoint therapy for melanoma metastatic to the brain: Investigating efficacy and the effects of relative treatment timing on lesion response. World Neurosurg..

[76] Ahmed K.A., Kim S., Arrington J., Naghavi A.O., Dilling T.J., Creelan B.C., Antonia S.J., Caudell J.J., Harrison L.B., Sahebjam S. (2017). Outcomes targeting the pd-1/pd-l1 axis in conjunction with stereotactic radiation for patients with non-small cell lung cancer brain metastases. J. Neurooncol..

[77] Chen L., Douglass J., Kleinberg L., Ye X., Marciscano A.E., Forde P.M., Brahmer J., Lipson E., Sharfman W., Hammers H. (2018). Concurrent immune checkpoint inhibitors and stereotactic radiosurgery for brain metastases in non-small cell lung cancer, melanoma, and renal cell carcinoma. Int. J. Radiat. Oncol. Biol. Phys..

[78] Lehrer E.J., Peterson J., Brown P.D., Sheehan J.P., Quinones-Hinojosa A., Zaorsky N.G., Trifiletti D.M. (2018). Treatment of brain metastases with stereotactic radiosurgery and immune checkpoint inhibitors: An international meta-analysis of individual patient data. Radiother. Oncol..

